# Diagnostic Usefulness of Disialotransferrin as an Indicator of Binge Drinking in Children and Adolescents

**DOI:** 10.3390/jcm13133833

**Published:** 2024-06-29

**Authors:** Bogdan Cylwik, Ewa Gruszewska, Katarzyna Janicka, Witold Olanski, Lech Chrostek

**Affiliations:** 1 Department of Paediatric Laboratory Diagnostics, Medical University of Bialystok, 15-274 Bialystok, Poland; kajanka@poczta.fm; 2 Department of Biochemical Diagnostics, Medical University of Bialystok, 15-269 Bialystok, Poland; ewa.gruszewska@umb.edu.pl (E.G.); lech.chrostek@umb.edu.pl (L.C.); 3 Department of Paediatric Emergency Medicine, Medical University of Bialystok Children’s Clinical Hospital, 15-274 Bialystok, Poland; wfakir@wp.pl

**Keywords:** binge drinking children and adolescents, transferrin isoforms, blood alcohol concentration

## Abstract

**Background/Objective:** The aim of the study was to evaluate the diagnostic usefulness of changes in transferrin isoforms, especially disialo-Tf, in identifying binge drinking children and adolescents admitted to hospital emergency. **Methods:** The study group consisted of 122 ambulatory children and adolescents below 18 years of age and 30 healthy subjects. From the group of drinkers, those with acute alcohol intoxication (AAI) were identified (ICD-11, code F10.0). The isoforms of transferrin were separated by capillary electrophoresis into five major fractions: asialo-Tf, disialo-Tf, trisialo-Tf, tetrasialo-Tf, and pentasialo-Tf. The differences between binge drinking youth and nondrinking subjects were evaluated by Mann–Whitney U-test. **Results:** In the total study group and in both genders, the concentration of disialo-Tf was significantly higher in the binge drinkers compared to the nondrinking youth (*p* = 0.006). With respect to the gender, the level of disialo-Tf was significantly higher in binge drinking than nondrinking girls (*p* = 0.028) and the value of trisialo-Tf was lower in binge drinking than nondrinking boys (*p* = 0.011). In the AAI subgroup, the concentrations of disialo-Tf and tetrasialo-Tf were significantly higher in comparison to nondrinking subjects (*p* = 0.002, *p* = 0.039, respectively). There were no significant correlations between the BAC and the transferrin isoforms in the total group and the AAI subgroup. The disialo-Tf reached the highest diagnostic power (AUC = 0.718) in identifying binge drinkers at diagnostic specificity and sensitivity of 86.7% and 51.6%, respectively (at cut-off 0.70), in the total group and it was growing up to AUC = 0.761 with the diagnostic sensitivity of 60% in the AAI subgroup. **Conclusions:** The disialo-Tf might be a useful biomarker to identify binge drinking children and adolescents.

## 1. Introduction

Alcoholism among young people is currently a very serious social problem in the world, as well as in Poland [1]. An increasing number of people start drinking for the first time at a very young age, between 12 and 16 years, when they are gaining more and more independence [2]. Many of them drink regularly until they become drunk. Alcohol addiction is also common, affecting younger and younger people. Within the group of drinkers, there are different patterns of alcohol consumption: those who drink moderately (so-called social drinkers), risky drinkers, those who become drunk (so-called binge drinkers), drink significant amounts of alcohol (so-called heavy drinkers), drink in a harmful way (so-called problem drinkers), and alcohol addicts (so-called alcoholics). An increasing number of children and adolescents with alcohol poisoning is admitted to hospital emergency departments; they are often found in the street and transported by ambulance, followed by observation and detoxification. The number of such interventions is rising [3]. As a rule, these are young people who drink large amounts of alcohol in a relatively short period of time, and in the drinking model they are classified as so-called binge drinking groups [4]. In this group, women consume 4 or more standard drinks, and men—5 or more drinks within a few hours per occasion [4]. Significant alcohol consumption in a short period of time among young people may cause serious side effects (hypothermia, reduced consciousness, metabolic acidosis, hypoglycemia) or, as a consequence, lead to somatic and psychological damage [5]. Among young people, there is a particular ease and tendency to progress from binge drinking to escalating abuse towards addiction. Adolescents from the so-called binge drinkers are currently the most dynamically growing group of alcohol abusers in the world. This group is dominated by so-called risky behaviors that lead to social and health damage. Therefore, there is an urgent need to identify this pattern of drinking among children and adolescents.

During chronic alcohol consumption, significant quantitative and qualitative changes occur in many biochemical blood parameters (e.g., disturbances of glycosylation/sialylation of proteins and lipids). We also expect alterations in the profile of transferrin isoforms, which could be used for diagnostic purposes and identification of minors at risk of alcoholism, e.g., in disialotransferrin that is one of 9 isoforms of transferrin containing 2 sialic acid residues in the carbohydrate chain of the protein [6]. It is part of the so-called low-carbohydrate transferrin CDT (carbohydrate-deficient transferrin) (with asialo- and monosialotransferrin), which is a common and routinely used marker of chronic alcohol abuse in adults and adolescents [7,8,9]. An increase in CDT concentration in serum usually occurs after regular consumption of 50–80 g of ethanol (5–8 standard drinks) per day for an uninterrupted period of at least 1 week, and the return to baseline values takes place slowly after about 2 weeks after cessation of drinking [10]. Disialotransferrin may also be an independent marker for detecting excessive alcohol consumption, which has been proven in the adult population [11,12,13]. The only report on research into disialotransferrin in adolescents (13–18 years of age) showed a significant increase in the concentration of this isoform (%DST) (HPLC method) in the group of binge drinkers compared to nonbinge drinkers [14]. Interestingly, such a difference did not occur in the case of CDT determination (N Latex CDT, immunonephelometric method).

Due to the fact that excessive alcohol consumption affects many metabolic processes in the liver, including protein glycosylation (sialylation), the aim of the study was to evaluate the diagnostic usefulness of changes in transferrin isoforms, especially disialotransferrin in identifying binge drinking children and adolescents admitted to hospital emergency departments.

## 2. Materials and Methods

### 2.1. Patients

**Figure 1:**
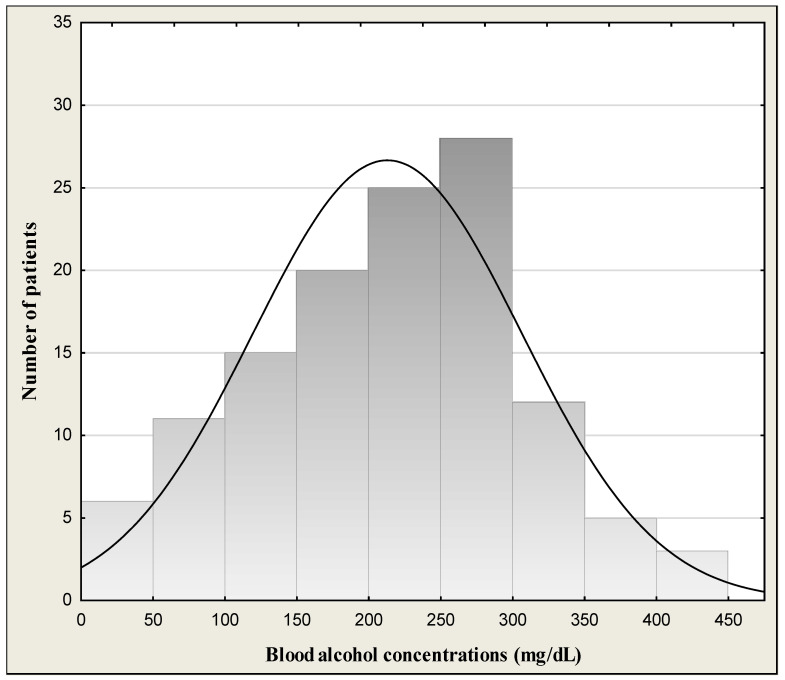
Blood alcohol concentration in the binge drinking youth.

**Figure 2:**
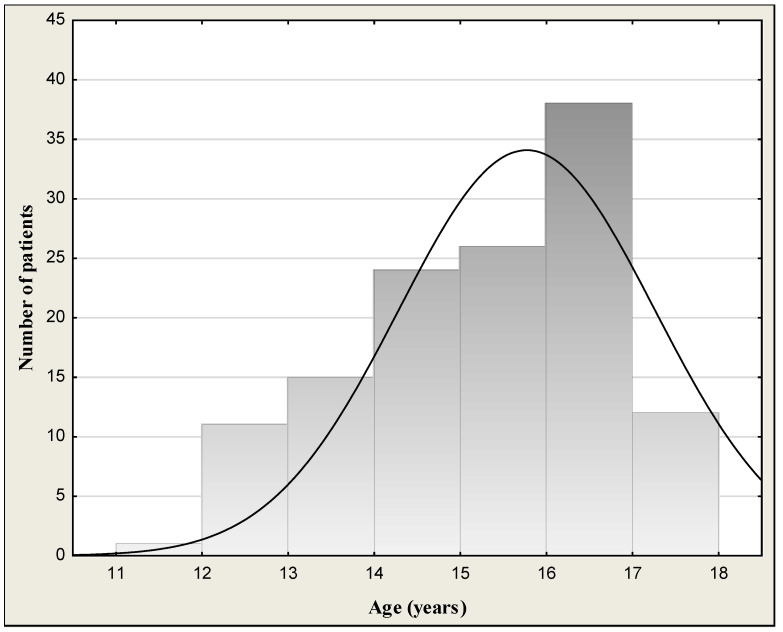
Age distribution of the binge drinking youth.

**Figure 3:**
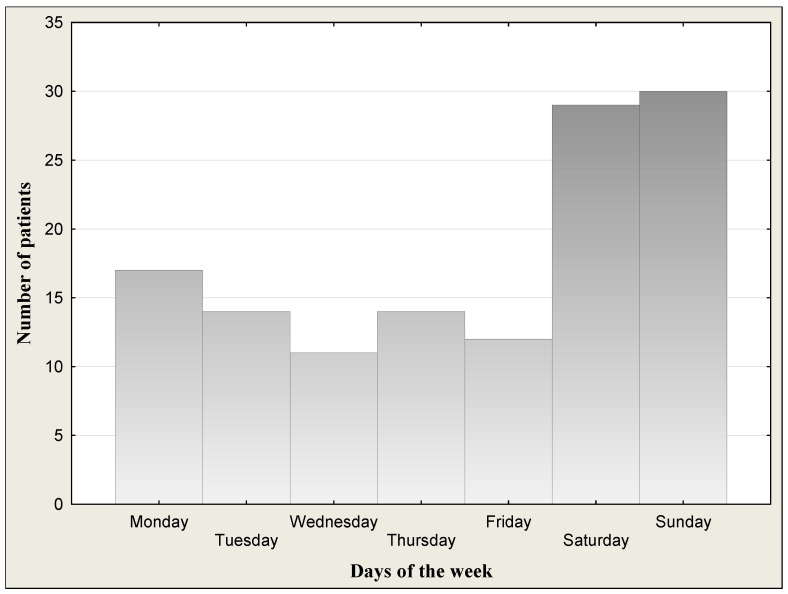
Days of the week of admission to hospital.

The study group consisted of 122 outpatient children and adolescents (62 girls and 60 boys) aged 12–18 (mean age 15.6 ± 1.59 years) successively reporting to physicians in the hospital emergency department of Children’s University Hospital in Bialystok due to alcohol poisoning. All patients underwent a physical examination. General condition of children was good or quite good and patients were cardiovascularly and respiratorily stable at admission to the hospital. Symptoms of the patients at admission are given in Table 1. They declared that it was a single incident of drinking a large amount of alcohol. Blood alcohol concentrations (BACs) in the binge drinking youth are presented in Table 2 and Figure 1. Data on distribution of age are given in Figure 2. Figure 3 shows the days of the week of admission to hospital. The majority of patients were admitted on weekends (Saturday–Sunday: 30 patients/day) in comparison to weekdays (Monday–Friday: 14 patients/day). Among all the patients admitted to hospital, a subgroup of 50 children and adolescents with acute intoxication (AAI) due to alcohol abuse (ICD-11 code F10.0) was identified [15]. Acute alcohol intoxication is a clinically harmful condition that usually follows the ingestion of a large amount of alcohol. The controls comprised 30 healthy children of both sexes (16 girls and 14 boys) from 10 to 18 years (mean age 14.7 ± 2.18) without alcohol intake, coming to hospital for complaints other than alcohol poisoning (headache, abdominal pain, chest, fainting, nausea and vomiting, behavioral disturbances, superficial injuries, others). The study was approved by the local research ethics committee for Medical University of Bialystok (APK.002.22.2020). Written informed consent was obtained from parents or guardians after explanation of the nature of the study.

### 2.2. Materials

Blood samples were taken from a peripheral vein, on admission day. The blood was allowed to completely clot at room temperature. The sera were separated by centrifugation at 1500× *g* for 5 min., collected, and stored at –80 °C until analysis. In addition to serum, a portion of each blood sample was collected into tubes containing EDTA-2 for hematological analysis. 

### 2.3. Methods

#### 2.3.1. Determination of Transferrin Isoforms

The isoforms of transferrin were separated by capillary electrophoresis on an MINICAP electrophoretic system using the MINICAP CDT reagent kits (Sebia, Evry, France) into five major fractions according to their sialylation level: asialotransferrin (asialo-Tf), disialotransferrin (disialo-Tf), trisialotransferrin (trisialo-Tf), tetrasialotransferrin (tetrasialo-Tf), and pentasialotransferrin (pentasialo-Tf). The system calculates relative concentrations (as a percentage of total transferrin) of each fraction automatically. The transferrin isoforms were separated by their electrophoretic mobility in an alkaline buffer with a specific pH 8.8.

#### 2.3.2. Biochemical Measurements

Total serum transferrin concentration was determined by the immunoturbidimetric method using the transferrin kit (TRSF2, Roche, Mannheim, Germany) on the Cobas 501c analyzer (Roche, Germany). In this method, specific antibodies to human transferrin form insoluble complexes and the result as a turbidity is measured on the analyzer. The concentration of alcohol in blood serum was determined using the enzymatic method with alcohol dehydrogenase on the Cobas 501 biochemical analyzer (Roche, Germany). The serum level of basic laboratory findings: C-reactive protein (CRP), alanine aminotransferase (ALT), and aspartate aminotransferase (AST) activity were determined using routine laboratory methods. The following reference values for children and adolescents were used: CRP: <5 mg/L; AST and ALT: 32 IU/L in girls and <40 IU/L in boys; AST/ALT ratio: <1.0.

#### 2.3.3. Hematological Assays

Blood cell counts were determined on the Sysmex XN-1000 analyzer (Sysmex, Kobe, Japan). The following reference values for children and adolescents were used: white blood cells (WBC): 3.98–10.04 10^3^/µL in girls and 4.23–9.07 10^3^/µL in boys; red blood cells (RBC): 3.93–5.22 10^6^/µL in girls and 4.63–6.08 10^6^/µL in boys; hemoglobin (Hb): 11.2–15.7 g/dL in girls and 13.7–17.5 g/dL in boys; hematocrit (Ht): 34.1–44.9% in girls and 40.01–51.0% in boys and mean corpuscular volume (MCV): 79.4–94.8 fL in girls and 79.0–92.2 fL in boys.

### 2.4. Statistics

Statistical analysis was performed using Statistica 13.3 PL (StatSoft, Kraków, Poland). The differences between binge drinking youth and nondrinking subjects were evaluated by Mann–Whitney U-test. The results were expressed as medians and ranges (lower Q1 and upper Q3 quartiles). The correlation between variables was assessed by Spearman’s rank correlation coefficient. The results were considered to be statistically significant when *p* values were less than 0.05. To calculate the diagnostic accuracy of transferrin isoforms profile in binge drinking youth, the area under the receiver operating characteristic (AUROC) curve was calculated. Diagnostic sensitivity, specificity, accuracy, and positive (PPV) and negative predictive values (NPV) were counted using optimal cut-offs that were determined according to the Youden method. 

## 3. Results

Demographic and laboratory characteristics of the study population are shown in Table 2. The median blood alcohol concentration (BAC) of binge drinking youth was 1.79‰ (mean: 1.73 ± 0.76‰; min.–max.: 0.48–3.44‰) or 220.5 mg/dL (mean: 212.9 ± 93.5 mg/dL; min.-max.: 59.1–423.8 mg/dL). We had 8 children with BAC < 50 mg/dL (approximate alcohol consumption 1–2 drinks), 10 children with BAC > 50 mg/dL (mild form of AAI) (approximately after 2–3 drinks), 33 children with BAC > 100 mg/dL (moderate form of AAI) (after 4–6 drinks), and 71 children with BAC > 200 mg/dL (severe form of AAI) (alcohol consumption 13–26 drinks). There was no statistically significant BAC difference between boys (median: 1.87‰; mean: 1.80 ± 0.79‰) and girls (median: 1.76‰; mean: 1.65 ± 0.73‰) (*p* = 0.439). The highest measured blood alcohol concentration was 3.44‰, attained by one boy of 17 years of age. The lowest BAC was 0.48‰ in girls (15 years). In turn, the youngest patient was a 12-year-old girl, and the oldest patients were 5 girls and 6 boys at the age of 18 (Figure 2). Figure 3 presents the day of the week of admission to hospital. The majority of patients were admitted to hospital on weekends (Saturday–Sunday: 30 patients/day) in comparison to weekdays (Monday–Friday: 14 patients/day). 

As for laboratory tests, among the biochemical findings, the median activity of serum AST was significantly elevated in drinking youth in comparison to the nondrinking youth (*p* = 0.024). AST and ALT activities in the blood may be increased after heavy consumption of alcohol and remain elevated after alcohol use for as long as 2–3 weeks [16]. In liver disease, when the ratio of serum AST to serum ALT is greater than two, then alcohol is likely to be contributing [17]. In our research, the ratio was normal (<2). 

Taking into account hematological parameters, the number of WBC and value of MCV were significantly higher in the study group than those in the controls (*p* < 0.001 for both comparisons), but the number of RBC was decreased (*p* = 0.048). There was significant correlation between the RBC and blood alcohol concentration (R = 0.202, *p* = 0.025). MCV is a long-term biomarker of alcohol consumption with timeframe to normalization 4 and more weeks; it elevates after 6 weeks of alcohol misuse and may remain elevated for up to 3 months after a person has stopped drinking [16,18]. High MCV (>100 fL) can be indicative of excessive alcohol use, about 60 g of alcohol per day [16]. Chronic and excessive drinking leads to enlarged red blood cells. 

**Figure 4:**
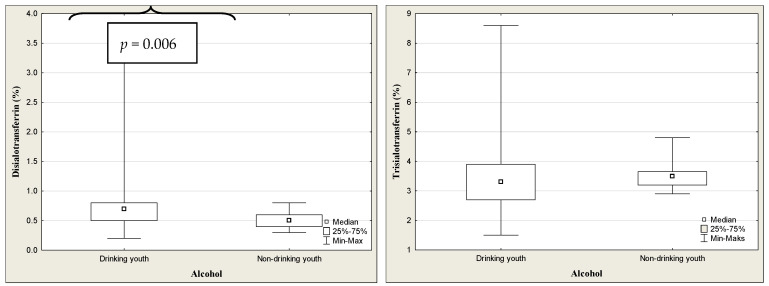

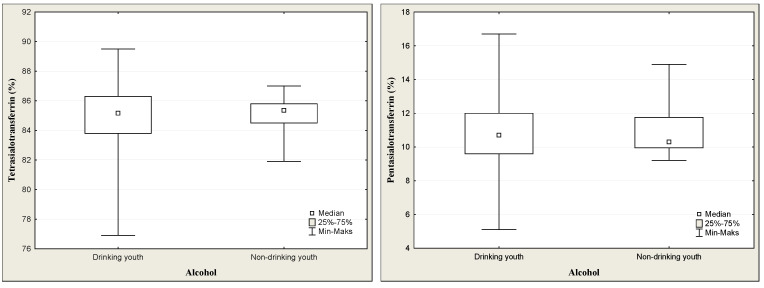
The concentrations of transferrin isoforms in binge drinking and nondrinking youth.

**Figure 5:**
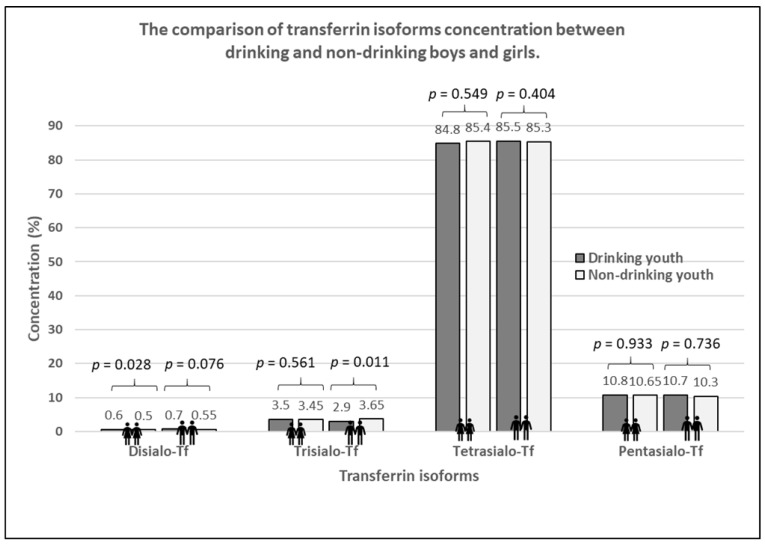
The comparison of transferrin isoforms in binge drinking and nondrinking boys and girls.

**Figure 6:**
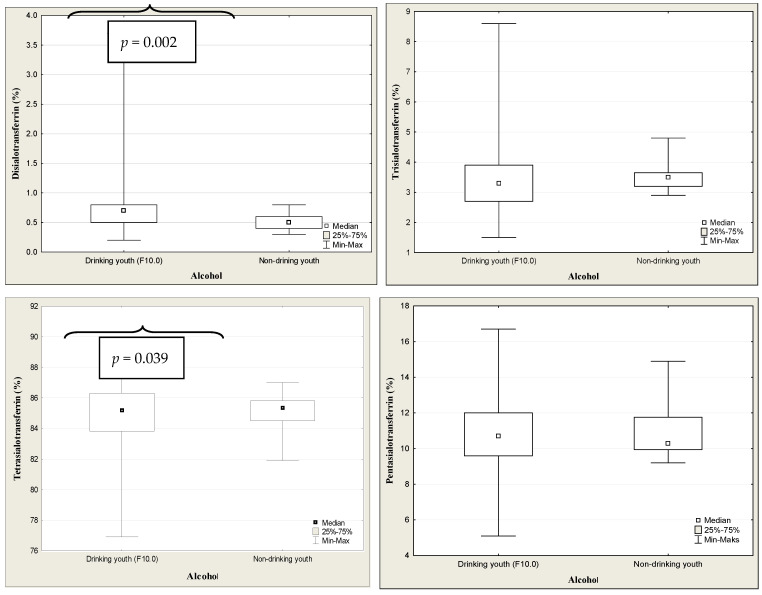
The concentrations of transferrin isoforms in children and adolescents with acute alcohol intoxication (F10.0) and nondrinking youth.

**Figure 7:**
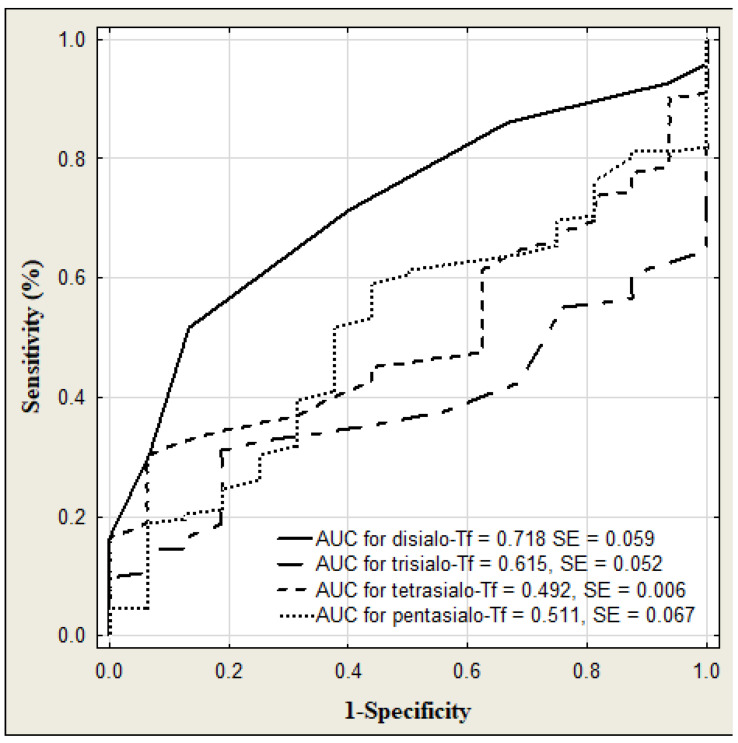
ROC curves for serum transferrin isoforms levels in binge drinking youth.

**Figure 8:**
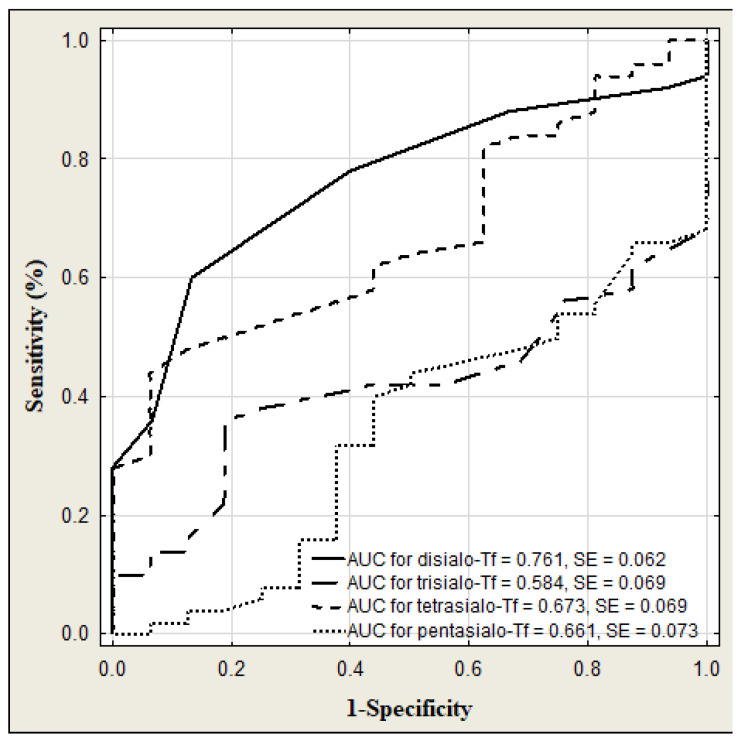
ROC curves for serum transferrin isoforms levels in children and adolescents with acute alcohol intoxication.

The results of transferrin and its isoforms in binge drinking youth are presented in Table 3 (Figure 4). The median of transferrin concentration was significantly higher in binge drinking youth compared to that in nondrinking subjects (*p* < 0.001). Among the transferrin isoform profile, only the concentration of disialo-Tf was significantly higher in the binge drinking youth compared to the nondrinking youth (*p* = 0.006). Taking gender into account, the medians of transferrin level were significantly higher in both the boys and girls groups in comparison to those in the nondrinking (3.05 versus 2.79%, *p* = 0.040; 3.27 versus 2.79%, *p* = 0.006). According to the gender, among the transferrin isoforms, only the median of disialo-Tf concentration in binge drinking girls was significantly higher than in the nondrinking girls (0.60 versus 0.50%, *p* = 0.028) (Figure 5). In turn, in the binge drinking boys, the value of trisialo-Tf was significantly lower than that in the nondrinking boys (2.90 versus 3.65%, *p* = 0.011). There were no significant correlations between the BAC and the transferrin isoforms: disialo-Tf (R = 0.141, *p* = 0.124), trisialo-Tf (R = −0.034, *p* = 0.710), tetrasialo-Tf (R = −0.023, *p*= 0.800) and pentasialo-Tf (R = 0.036, *p* = 0.695). In turn, Table 4 and Figure 6 show the results of transferrin isoforms in the subgroup of children and adolescents selected due to the acute alcohol intoxication (ICD-11 code F10.0). There were slight differences between the entire drinking group and those with acute alcohol poisoning. In this subgroup, the median of transferrin level was also significantly higher in the binge drinking youth than that in the nondrinking subjects (*p* < 0.001). However, in the profile of transferrin isoforms, in contrast to the entire drinking population, the concentrations of two isoforms were significantly increased: disialo-Tf and tetrasialo-Tf in comparison to the nondrinking subjects (*p* = 0.002, *p* = 0.039, respectively). In this subgroup, there were no significant correlations between the BAC and the isoform transferrin profile: disialo-Tf (R = 0.100, *p* = 0.489), trisialo-Tf (R = 0.086, *p* = 0.550), tetrasialo-Tf (R = −0.048, *p* = 0.738), and pentasialo-Tf (R = −0.012, *p* = 0.935). The diagnostic power of transferrin isoforms is presented in Table 5 and Table 6 and Figure 7 and Figure 8. Among the transferrin isoforms, disialo-Tf has the highest diagnostic power (AUC = 0.718) in the entire binge drinking youth group (Figure 7, Table 5). This isoform had also the highest diagnostic specificity (86.7%) at the lowest diagnostic sensitivity (51.6%) in binge drinking children. In turn, as the study group was standardized, i.e., the children and adolescents with acute alcohol intoxication, the diagnostic power of disialo-Tf was growing up to the area under the curve (AUC) = 0.761 at the same diagnostic specificity (86.7%) and a higher diagnostic sensitivity (60%) (Figure 8, Table 6). The diagnostic powers of two other isoforms, tetrasialo-Tf and pentasialo-Tf, were AUC = 0.673 and AUC = 0.661, respectively. It was observed that the tetrasialo-Tf had the highest diagnostic specificity (93.8%) and the lowest diagnostic sensitivity (44%), contrary to pentasialo-Tf, which had the highest diagnostic sensitivity (100%) and the lowest diagnostic specificity (32%) (Figure 8, Table 6).

## 4. Discussion

Alcohol is the oldest, toxic, and most diffusive psychoactive and dependence-producing substance used in the world. Its excessive consumption can have a severe and detrimental impact on society. Heavy drinking can increase the risk of physical and mental health complications. About 90% of people have contact with alcohol at various stages in their lives, and up to 30% of them will develop alcohol-related disorders [19,20]. The problem is that alcohol consumption increases with age. A growing number of people start drinking for the first time at a very young age, between 12 and 16 years, when they are gaining more and more independence. Many of them drink regularly until they become drunk. As a rule, these are young people who drink large amounts of alcohol in a relatively short period of time, and in the drinking model they are classified as so-called binge drinking groups [4]. One of the most frequent alcohol-related disorders present in patients admitted to hospital emergency departments is acute alcohol intoxication that occurs not only in adults but also in children and adolescents [21]. 

Consumption of large amounts of alcohol is associated with numerous metabolic changes in the liver. For example, alcohol misuse impairs the glycosylation, secretion, and metabolism of many glycoproteins, including transferrin, which may affect their blood concentration. These quantitative alterations may accompany the qualitative changes in the form of glycosylation disturbance, which is documented by the increased level of low-sialylated isoforms of transferrin. Patients with chronic alcohol consumption may develop abnormally glycosylated transferrin isoforms. Carbohydrate-deficient transferrin (CDT) is a sum of three low-sialylated isoforms, asialo-Tf, monosialo-Tf, and disialo-Tf, and is widely used to detect and monitor alcohol misuse [22]. A regular intake of 50–80 g ethanol per day for a minimum of 1–2 weeks induces transferrin microheterogeneity by increasing CDT isoforms [23,24]. Upon stopping alcohol intake, the CDT concentration returns to normal after two to four weeks. Based on the information on impaired glycosylation of glycoproteins during chronic alcohol abuse in adults, we expect that these abnormalities may occur in young people who drink large amounts of alcohol but in a short period of time [25,26,27,28]. Therefore, the aim of the study was to evaluate the diagnostic usefulness of transferrin isoforms in identifying drinking youth, especially binge drinking children and adolescents admitted to hospital emergency departments. To our knowledge, this is the first study conducted to examine the diagnostic usefulness of the profile of transferrin isoforms in identifying binge drinking adolescents younger than 18 years.

The group of drinking youth in our study may be diverse in terms of alcohol consumption, as we do not have detailed information concerning drinking history. The group could include teenagers who are heavy drinkers and regular drinkers (chronic drinking), as well as those who drink large amounts of alcohol only occasionally in a relatively short period of time (binge drinking). However, based on the normal MCV value and AST/ALT ratio (markers of chronic drinking), it should be assumed that our study group of drinking youth did not include chronic long-time alcohol drinkers. The fact that the majority of drinkers were admitted to the ward on weekends may indicate that they belonged to the binge drinking group. We observed that twice as many drinking adolescents were admitted to hospital on weekends (Saturday–Sunday) than during the week (Monday–Friday). Binge drinking is defined as a pattern of drinking that brings blood alcohol concentration (BAC) levels to 80 mg/dL or above. This typically occurs after consuming four or more drinks for women and five or more drinks for men, in about 2 h per occasion [4]. In our study, the range of BAC) was wide and ranged from 59.1 to 423.8 mg/dL with median of 220.5 mg/dL. It means that children could drink at least 8 beers (500 mL), 2 bottles of wine (750 mL), or 1 bottle of vodka (500 mL). Beer is still the most commonly chosen alcoholic drink [29,30]. The elevation of BAC is useful to confirm the suspicion of intoxication [31]. In binge drinking patterns, alcohol is most often consumed less than once a week or once a week [29]. Binge drinking is the pattern of alcohol consumption most correlated with acute alcohol intoxication (AAI). A European standard single drink contains about 10–12 g of ethanol, which is estimated to increase the blood alcohol concentration of a 70 kg man by 15–20 mg/dL. A standard drink represents the quantity of alcohol contained approximately in a 0.33 cl beer, a standard glass of red wine (125 mL), or a shot of spirit (40 mL) [32].

Our study revealed a shift in the profile of transferrin isoforms in the binge drinking adolescents. In binge drinking youth, this change involves an increase in low-sialylated fraction such as disialo-Tf as compared to the nondrinking individuals. Interestingly, if the study group is divided by gender, only the girls show an increase in the concentration of disialo-Tf, while in the boys a decrease is observed in another fraction—trisialo-Tf. As we know, alcohol consumption in adolescents increases with age, with girls showing the greatest increase. We have to remember that women are more susceptible to alcohol toxicity as their bodies contain more adipose tissue but less body fluid than men, which leads to greater concentrations of alcohol within the circulation [33]. Moreover, women possess 70–80% less alcohol dehydrogenase than men, which is an enzyme responsible for metabolizing alcohol. It seems that greater alcohol consumption by girls and its delayed metabolism as compared to men are important factors that may more effectively lead to a shift in the profile of transferrin isoforms towards low-sialylated fraction, like disialo-Tf. Stokbroekx et al. showed significantly higher disialo-Tf concentration in binge drinkers in comparison to nonbinge drinkers measured by HPLC method [14]. These children consumed 80 g of alcohol at intoxication or 60 g in month before intoxication [14]. Acute alcohol intoxication additionally shows an increase in tetrasialo-Tf level. In the population of alcohol abusers, two mechanisms are proposed to be responsible for the increase in the concentration of desialylated isoforms of glycoproteins, namely, a decreased activity of cellular glycosylotransferases (i.e., mannosylotransferase, galactosylotransferase, N-acetyl-glucosaminylotransferase, sialyltransferase) and an increased serum activity of sialidase [34,35]. There were no correlations between the BAC and the isoform transferrin profile in binge drinking youth as well as in the AAI group. Taking into account gender in the study drinking group, we observed higher concentrations of trisialo-Tf and tetrasialo-Tf in girls but lower in the boys. The same comparison in the control nondrinking group showed no differences in transferrin isoforms due to gender. This may undoubtedly indicate that alcohol impairs transferrin glycosylation in children who persistently drink large amounts of alcohol in a short period of time, with girls being more at risk. We found that among all transferrin isoforms, disialo-Tf had the highest diagnostic power (the fair diagnostic power) in identifying binge drinking children and adolescents. This value was growing in the acute alcohol intoxication (good diagnostic power).

The main limitations of the study are the small sample size and the lack of data on drinking history. Therefore, further studies are needed. We had no data on alcohol consumption comprehended as the amount of alcohol consumed on the day of intoxication and the amount of alcohol consumed in the month before admission to hospital emergency department.

## 5. Conclusions

In summary, the results of our study indicate that the disialo-Tf assay might be a useful biomarker to detect binge drinking in all children and adolescents and that both isoforms, disialo-Tf and tetrasialo-Tf, might be useful to recognize binge drinking in children and adolescents with acute alcohol intoxication.

## Figures and Tables

**Table 1:** Clinical characteristics of the study population.

Symptoms of Patients	Number of Patients
Impaired consciousness	115
Somnolence	98
Nausea and vomiting	75
Vertigo	54
Comatose	8
Aggressive behavior	7
Disorientation	6
Hypothermia	5

**Table 2:** Demographic and laboratory characteristics of the study population.

	Binge Drinking Youth (*n* = 122)	Nondrinking Youth (*n* = 30)	*p* Value *
Age (years) (median/range)	16 (12–18)	15 (10–18)	-
Gender (girls/boys)	62/60	16/14	-
BAC (‰) BAC (mg/dL)	1.79 1.21–2.25 220.5 149.1–277.2	ND ND	- -
WBC (×10^3^/µL)	7.95 6.60–10.65	6.40 5.42–7.21	*p* < 0.001
RBC (×10^12^/L)	4.72 4.38–5.05	4.86 4.53–5.26	*p* = 0.048
Hb (g/L)	13.8 12.8–14.7	13.2 12.7–14.0	NS
Ht	40.3 37.0–42.5	39 36.6–40.9	NS
MCV (fL)	83.7 82.0–85.7	80.5 77.2–82.5	*p* < 0.001
PLT (10^9^/L)	264.5 226–307	266 227–310	NS
CRP (mg/L)	0.60 0.30–1.05	0.43 0.30–2.67	NS
AST (IU/L)	22.0 19.0–27.0	18.5 10.0–26.0	*p* = 0.008
ALT (IU/L)	13.0 11.0–19.0	13.0 10.0–20.0	NS
AST/ALT (ratio)	1.61 1.31–2.00	1.40 0.91–2.00	NS

Data are presented as medians and quartiles (Q1 and Q3). The differences between young drinkers and nondependent controls group were estimated by Mann–Whitney U-test. Significant difference at *p* < 0.05. * When comparing study group and controls. Abbreviations: BAC, blood alcohol concentration; WBC, white blood cells; RBC, red blood cells; Hb, hemoglobin; Ht, hematocrit; MCV, mean corpuscular volume; PLT, platelets; CRP, C-reactive protein; AST, aspartate aminotransferase; ALT, alanine aminotransferase; ND, not detectable; NS, not significant.

**Table 3:** The results of transferrin isoforms in binge drinking and nondrinking youth.

Group	Tf (g/L)	Disialo-Tf (%)	Trisialo-Tf (%)	Tetrasialo-Tf (%)	Pentasialo-Tf (%)
Binge drinking youth (*n* = 122)	3.10 2.85–3.41	0.70 0.50–0.80	3.30 2.70–3.90	85.1 83.8–86.3	10.7 9.60–12.0
Nondrinking youth (*n* = 30)	2.76 2.48–3.00 *p* < 0.001	0.50 0.40–0.60 *p* = 0.006	3.50 3.20–3.65 *p* = 0.134	85.3 84.5–85.8 *p* = 0.918	10.3 9.95–11.7 *p* = 0.886

Data are medians and quartiles (Q1 and Q3). The differences between young drinkers and nondependent controls group estimated by Mann–Whitney U-test. Significant difference at *p* < 0.05. Abbreviations: Tf, transferrin.

**Table 4:** The results of transferrin isoforms in children and adolescents with acute alcohol intoxication (F10.0) and nondrinking youth.

Group	Tf (g/L)	Disialo-Tf (%)	Trisialo-Tf (%)	Tetrasialo-Tf (%)	Pentasialo-Tf (%)
Acute alcohol intoxication (*n* = 50)	3.10 2.85–3.41	0.70 0.60–0.90	3.30 2.60–3.90	85.8 84.8–87.2	10.0 8.80–10.7
Nondrinking youth (*n* = 30)	2.76 2.48–3.00 *p* < 0.001	0.50 0.40–0.60 *p* = 0.002	3.50 3.20–3.65 *p* = 0.320	85.3 84.5–85.8 *p* = 0.039	10.3 9.95–11.7 *p* = 0.054

Data are medians and quartiles (Q1 and Q3). The differences between young drinkers and nondependent controls group estimated by Mann–Whitney U-test. Significant difference at *p* < 0.05. Abbreviations: Tf, transferrin.

**Table 5:** The diagnostic power of transferrin isoforms profile in binge drinking youth.

	Cut-off (%)	Sensitivity (%)	Specificity (%)	ACC (%)	PPV (%)	NPV (%)	AUC ± SE
Disialo-Tf	0.70	51.6	86.7	55.5	96.9	18.1	0.718 ± 0.059
Trisialo-Tf	2.90	100	35.2	42.8	16.8	100	0.615 ± 0.052
Tetrasialo-Tf	83.7	93.8	21.3	29.7	13.5	96.3	0.492 ± 0.006
Pentasialo-Tf	10.5	59.0	56.3	58.7	91.1	15.3	0.511 ± 0.067

ACC diagnostic accuracy, PPV positive predictive value, NPV negative predictive value, AUC area under ROC curve, SE standard error.

**Table 6:** The diagnostic power of transferrin isoforms profile in children and adolescents with acute alcohol intoxication (F10.0).

	Cut-off (%)	Sensitivity (%)	Specificity (%)	ACC (%)	PPV (%)	NPV (%)	AUC ± SE
Disialo-Tf	0.70	60.0	86.7	66.2	93.8	39.4	0.761 ± 0.062
Trisialo-Tf	2.90	100	32.0	48.5	32.0	100	0.584 ± 0.069
Tetrasialo-Tf	86.2	44.0	93.8	56.1	95.7	34.9	0.673 ± 0.069
Pentasialo-Tf	9.20	100	32.0	48.5	32.0	100	0.661 ± 0.073

ACC diagnostic accuracy, PPV positive predictive value, NPV negative predictive value, AUC area under ROC curve, SE standard error.

## Data Availability

The raw data supporting the conclusions of this article will be made avaiable by the authors on request.
